# Dr. Jacob M. Kraus

**Published:** 1903-01

**Authors:** 


### OBITUARY.

Dr. Jacob M. Kraus, of Buffalo, U. of B., '89, died at his home in this city December 19, 1902, aged 36 years. Dr. Kraus had been ill for several months, and went to Denver in the autumn with the hope of recuperation, but this proved a disappointing hope and he returned a few weeks ago. He had been steadily failing until death came as stated. His disease proved to be intestinal tuberculosis. He was one of the brightest of the younger physicians of Buffalo, and was always found upon the side of professional integrity and business honor. He was a native of Clarence, N. Y., and a graduate of the Academy at that place. He is survived by a widow and two children, aged nine and five years respectively, the elder being a boy and the younger a girl.

The Roswell Park Medical Club, at a special meeting, adopted the following memorial:

For the first time in the history of the club we are called upon to mourn the death of one of our members.

Dr. Jacob M. Kraus died at his home in this city December 19, 1902, at the age of 36 years. In his death the club loses a most esteemed member and well-beloved associate.

Dr. Kraus was always modest and unassuming, courteous in demeanor and genial in companionship—in all respects a model gentleman. His principles were as fixed as the planets—he was what the poet calls "the noblest work of God, an honest man." His skill as a physician was unquestioned; his professional councils always weighed with his associates.

As son, brother, husband and father, he did his whole duty and did it cheerfully. He will be missed by all who knew him —further than this eulogy cannot go.

The entire experiences of Dr. Kraus were marked by uncommon difficulties and hardships, but buoyed by a heroic will and strengthened by a conscientious purpose, he pushed steadily on until he was able to overcome them all. He had just begun to reap where he had so persistently and generously sown, when he himself was gathered in by "the reaper whose name is death."

The club associates of Dr. Kraus unite in expressing their deep grief at his "untimely taking off" and their truest sympathy with the family so suddenly and sorely bereaved.

The medical staff of the Dispensary of the German Hospital, at a special meeting held December 19, 1902, adopted the following memorial and resolutions:

The news of the untimely death of our friend and colleague, Dr. Jacob M. Kraus, fills us with unspeakable sorrow. Dr. Kraus has been a member of the staff of this dispensary since its inception, and until prevented by sickness from continuing his service has shirked no duty, but discharged every obligation faithfully and well.

His untiring labors, his studious habits and his practical accomplishments had already borne the fruit of a large practice, and his manly virtue and professional integrity have endeared him to all who knew him.

Dr. Kraus was one of nature's noblemen, a man of fidelity, integrity and honesty. What greater praise can be paid to any mortal?

*Resolved*, That we express to the family of Dr. Kraus our sincerest sorrow.

*Resolved*, That this memorial and these resolutions be spread on the minutes, a copy sent to the family and copies to the medical press.

*Resolved*, That we attend the funeral in a body.

CHARLES J. MENGIS, M. D.,

ABRAM L. WEIL, M. D.,

CHARLES WEIL, M. D.

} *Committee.*

